# Geografie und Gesundheit – das Beispiel der COVID-19-Pandemie in Bremen

**DOI:** 10.1007/s00103-025-04125-2

**Published:** 2025-09-04

**Authors:** Christoph Buck, Daniela Koller, Eva Kibele, Katharina Schulze, Jobst Augustin

**Affiliations:** 1 Leibniz Institut für Präventionsforschung und Epidemiologie – BIPS, Bremen, Deutschland; 2 Institut für Medizinische Informationsverarbeitung, Biometrie und Epidemiologie, Ludwig-Maximilians-Universität München (LMU), München, Deutschland; 3 Statistisches Landesamt Bremen, Bremen, Deutschland; 4 Institut für Versorgungsforschung in der Dermatologie und bei Pflegeberufen (IVDP), Universitätsklinikum Hamburg-Eppendorf (UKE), Martinistraße 52, 20246 Hamburg, Deutschland

**Keywords:** Raum, Epidemiologie, Infektionserkrankung, Stadtgesundheit, Deutschland, Space, Epidemiology, Infectious disease, Urban health, Germany

## Abstract

**Hintergrund:**

Gegenstand der Geografie ist unter anderem die Analyse raumzeitlicher Veränderungen von Strukturen und Prozessen. Die Gesundheitsgeografie wendet Methoden, Modelle und Paradigmen der Geografie auf gesundheitsspezifische Fragestellungen an. Am Beispiel der COVID-19-Pandemie in Bremen soll die geografische Perspektive auf Gesundheit aufgezeigt sowie deren Nutzen verdeutlicht werden.

**Methoden:**

Grundlage der Untersuchung sind raumzeitliche Daten der COVID-19-Neuinfektionen nach Kalenderwoche auf Ortsteilebene in der Stadt Bremen zwischen März 2020 und Mai 2022. Neben den Fallzahlen wurden zur Erklärung dieser ausgewählte Indikatoren zur soziodemografischen Lage (z. B. Haushaltsstruktur, Sozial- und Migrationsstatus) berücksichtigt. Die raumzeitlichen Analysen erfolgten deskriptiv sowie unter Verwendung linearer Regressionsmodelle.

**Ergebnisse:**

Die erste Pandemiewelle zeigt deutliche lokale Unterschiede und hohe Inzidenzen bzw. Periodenprävalenzen in einzelnen Ortsteilen. Für die späteren Wellen konnte eine Clusterbildung mit hohen Fallzahlen in vorwiegend deprivierten Ortsteilen identifiziert werden. Beispielsweise zeigt sich in der 2. Welle u. a. eine Assoziation zwischen den Fallzahlen und der Anzahl der Personen pro Haushalt (β = 1,099, *p* < 0,001), in der 4. Welle mit der Quote von Bürgergeldempfängern nach dem zweiten Sozialgesetzbuch (SGBII; β = 0,056, *p* = 0,004).

**Diskussion:**

Die Ergebnisse zeigen räumliche Unterschiede in den COVID-19-Fallzahlen und eine stärkere Belastung von deprivierten Ortsteilen. Die Untersuchung hat den hohen Nutzen einer raumzeitlichen Perspektive, hier am Beispiel der COVID-19-Pandemie in Bremen, aufgezeigt. Dies betrifft nicht nur die Analyse der Pandemiedynamik, sondern auch aus Public-Health-Perspektive die Identifizierung vulnerabler Bevölkerungsgruppen sowie die gezielte Implementierung von Präventionsmaßnahmen.

## Abstract (en)

**Background:**

Geography is, among other things, the study of spatial and temporal changes in structures and processes. Health geography applies the methods, models, and paradigms of geography to health-related issues. The example of the COVID-19 pandemic in Bremen is used to illustrate the geographical perspective on health and its benefits.

**Methods:**

The study is based on spatio-temporal data of new COVID-19 infections by calendar week at the district level in the city of Bremen between March 2020 and May 2022. In addition to the number of cases, selected indicators of the socio-demographic situation (e.g., household structure, social status, and migration status) were taken into account. Spatio-temporal analyses were performed descriptively and using linear regression models.

**Results:**

The first wave of the pandemic shows clear local differences and high incidence respectively period prevalence in individual city districts. For the later waves, a clustering with high case numbers in predominantly deprived city districts was identified. For example, in wave 2 there was an association between the number of cases and the number of persons per household (β = 1.099, *p* < 0.001) and in wave 4 with the SGBII rate (β = 0.056, *p* = 0.004).

**Discussion:**

The results show spatial differences in COVID-19 case numbers and a greater burden in deprived city districts. The study has shown the great benefit of a spatio-temporal perspective using the example of the COVID-19 pandemic in Bremen. This applies not only to the analysis of the dynamics of the pandemic, but also from a public health perspective to the identification of vulnerable populations and the implementation of targeted prevention measures.

## Einleitung

Die Geografie ist eine allumfassende Disziplin, die ein Verständnis der Erde und ihrer menschlichen sowie natürlichen Komplexität anstrebt – sie fragt nicht nur danach, wo sich Objekte befinden, sondern auch, wie sie sich verändert haben und entstanden sind [1]. Diese Beschreibung beinhaltet zum einen den klassischen räumlichen Bezug der Geografie auf unterschiedlichen Maßstabsebenen, zum anderen auch die zeitliche Dimension basierend auf der Frage von Entstehung und Veränderung von Prozessen. Nach Gebhard et al. kann die Geografie aufgrund ihrer Tätigkeitsfelder als Natur- und Gesellschaftswissenschaft angesehen werden, da sie sowohl die natürlichen (z. B. Veränderung von Landschaftsformen) als auch die gesellschaftlichen (z. B. sozialräumliche Veränderungen) Phänomene [2] unter Berücksichtigung empirischer Methoden und theoretischer Ansätze untersucht. Die Geografie kann aufgrund ihrer thematischen Spannweite sowie ihres holistischen Ansatzes auch als „Brücke zwischen den Natur- und Sozialwissenschaften“ bezeichnet werden [3].

Die Gesundheitsgeografie als Disziplin zwischen Geografie und Medizin bzw. Gesundheitswissenschaften beinhaltet die Anwendung geografisch-wissenschaftlicher Methoden, geografischer Modelle und Paradigmen, die auf spezifische Fragestellungen aus Medizin, Epidemiologie und insbesondere Public Health zurückgreift. Sie untersucht vor allem Raummuster, räumliche Prozesse und Mensch-Umwelt-Beziehungen im Kontext von Gesundheit und Krankheit [4]. Dazu gehören beispielsweise die Themenfelder räumliche Epidemiologie, regionale Versorgungsforschung (inkl. Zugang und Erreichbarkeit von Gesundheitsdienstleistungen), gesundheitsfördernde (therapeutische) Landschaften oder auch Gesundheit und Entwicklung. Hervorzuheben ist die hohe interdisziplinäre Ausrichtung der Gesundheitsgeografie, etwa mit Fächern der Sozial-, (z. B. Soziologie und Demografie), Gesundheits- (z. B. Epidemiologie) oder auch Naturwissenschaften (z. B. Meteorologie).

Einer der zentralen Aspekte der Gesundheitsgeografie ist die Frage nach räumlichen Unterschieden von Gesundheit und Krankheit und den zugrunde liegenden Ursachen und Auswirkungen. Regionale Aspekte werden dabei unterschieden in Effekte der Bevölkerungszusammensetzung (kompositionelle Effekte), Effekte des kleinräumigen Kontexts (kontextuelle Effekte) sowie Selektionseffekte [4]. Kompositionelle Effekte beruhen auf den aggregierten Merkmalen der Individuen, die in einer bestimmten Region leben, wie beispielsweise Alter, Geschlecht, Sozialstatus oder Gesundheitsstatus. Hingegen beschreiben kontextuelle Effekte die Merkmale des Raumes, wie etwa das Lärmaufkommen, Vorhandensein von Grünflächen oder das mittlere Einkommen (unabhängig von den Individualmerkmalen der Bewohner). Kontextuelle Faktoren unterstreichen die Bedeutung des Ortes und sind Abbild für gesundheitliche Ungleichheiten, da ortsbezogene Gelegenheitsstrukturen (materielle und soziale Rahmenbedingungen) Gesundheit und Gesundheitspraktiken fördern oder hemmen können. Schließlich können Selektionseffekte nach Andrews und Moon [5] das Resultat eines Migrationsprozesses beschreiben, bei dem beispielsweise Menschen mit besserem Gesundheitsstatus häufiger in kompositionell und kontextuell privilegiertere Quartiere ziehen.

Es gibt zahlreiche konzeptionelle Ansätze zur Erklärung kleinräumiger Merkmale von Gesundheit [6–9]. Exemplarisch genannt werden soll an dieser Stelle Mielck [7], der zwischen den Ansätzen unterscheidet, die sich auf sozioökonomische Prädiktoren (Armut, Bildungsungleichheit) konzentrieren, und denen, die Umweltrisiken betrachten (Umweltgerechtigkeit).

Im Zusammenhang mit Gesundheit lassen sich mehrere räumliche Dimensionen unterscheiden, von der Makro- bis zur Mikroebene [10]. Im besonderen Fokus stehen die kleinräumigen Ebenen wie statistische Bezirke oder Quartiere, die zur Identifizierung kleinräumiger regionaler Gesundheitsunterschiede gut geeignet sind [11].

Neben der Nutzung kartografischer Methoden zur explorativen Datenanalyse und Kommunikation (z. B. im öffentlichen Gesundheitsdienst) ist die Anwendung sog. Geoinformationssysteme (GIS), einhergehend mit Methoden der räumlichen Datenanalyse (z. B. raumzeitliche Cluster- oder Regressionsanalysen), von hoher Bedeutung für die gesundheitsgeografische Forschung [12]. Mittels dieser Verfahren lassen sich relevante Einflussgrößen auf Gesundheit und Krankheit in ihrer raumzeitlichen Dynamik erfassen, analysieren und visualisieren.

Zusammenfassend lässt sich sagen, dass die Berücksichtigung der räumlichen Dimension, sei es als geometrischer Raum (Space) oder als bedeutungsgeladener Ort (Place; [13]), in Verbindung mit der Anwendung einschlägiger qualitativer (v. a. zu Place) und quantitativer Methoden zur Analyse sowie einer holistischen Perspektive auf Gesundheit und Krankheit als Stärke und Alleinstellungsmerkmal der Gesundheitsgeografie angesehen wird [12].

Die COVID-19-Pandemie hat den hohen Mehrwert und die Notwendigkeit einer raumzeitlichen Betrachtung auf Gesundheit deutlich aufgezeigt. Im internationalen Kontext haben dies zahlreiche Studien verdeutlicht, die sowohl Determinanten bzw. Risikofaktoren [14, 15], Methoden [16, 17] oder auch Pandemieverläufe auf nationaler [18] oder kleinräumiger innerstädtischer [19, 20] Ebene untersucht haben. Im internationalen Vergleich wird jedoch deutlich, dass die Studienlage für Deutschland dazu immer noch relativ gering ist [21]. Das betrifft insbesondere die kleinräumige (innerstädtische) Betrachtung des Pandemiegeschehens, wenngleich sich Schmitz et al. am Beispiel Berlin-Neukölln [22] oder Schmiege et al. für die Stadt Essen [21] auf kleinräumiger Ebene mit der Pandemie befasst haben. Schmiege et al. konnten dabei feststellen, dass eine Assoziation von bebauter Umwelt und der Ausbreitung von SARS-CoV-2-Infektionen besteht, da geräumigere Wohnungen oder ein höheres Maß an städtischem Grün mit niedrigeren Infektionsraten in Nachbarschaften innerhalb der Stadtteilebene assoziiert sind. Die ungleiche innerstädtische Verteilung dieser Faktoren unterstreicht die vorherrschenden umweltbedingten gesundheitlichen Ungleichheiten im Zusammenhang mit der COVID-19-Pandemie, so die Autor:innen. Am Beispiel von Berlin-Neukölln konnten Schmitz et al. zeigen, dass die Maßnahmen zur Kontaktreduzierung einen erheblichen Einfluss auf die Inzidenz hatten und sich die Hochrisikogebiete räumlich über die Pandemiephasen hinweg verändert haben. Mit Blick auf die Sozialstruktur zeigte sich bundesweit in den ersten Wellen, dass beispielsweise Gemeinden in strukturschwachen Regionen stärker von erhöhten COVID-19-Infektions- und Mortalitätsraten betroffen waren, unter anderem aufgrund einer höheren Prävalenz chronischer Erkrankungen [23]. In einer umfangreichen Variablenselektion von Statistiken zur Bebauung, Umwelt und Deprivation auf Gemeindeebene in Deutschland identifizierten Scarpone et al. [24] zum Beispiel die Infrastruktur und sozioökonomische Faktoren als wichtige Prädiktoren für COVID-19-Inzidenzraten während der 1. Welle.

In Ergänzung zu den wenigen existierenden nationalen Studien verfolgt die vorliegende Untersuchung 2 übergeordnete Ziele: 1) kleinräumige längsschnittliche Analyse und Charakterisierung des Pandemiegeschehens am Beispiel der Hansestadt Bremen, 2) Verdeutlichung des Nutzens einer raumzeitlichen Betrachtung des Krankheitsgeschehens am Beispiel von COVID-19.

## Methoden

Für die räumlichen Analysen des Verlaufs der COVID-19-Infektionen in der Stadt Bremen wurden im Rahmen eines DFG-geförderten Projektes (BU 4156/2-1) 2 Datensätze auf räumlicher Ebene der Stadtteile verknüpft. Die raumzeitlichen Daten umfassten die COVID-19-Neuinfektionen pro Kalenderwoche (KW) in 89 Ortsteilen der Stadt Bremen und wurden von der Senatorin für Gesundheit, Frauen und Verbraucherschutz der Freien Hansestadt Bremen zur Verfügung gestellt. Der Infektionsverlauf wurde zwischen dem Beginn der Pandemie im März 2020 bis Mai 2022 beobachtet und enthielt somit Daten über verschiedene Wellen der Pandemie. Aus Datenschutzgründen wurden COVID-19-Inzidenzen für Ortsteile mit weniger als 100 Einwohnern sowie mit weniger als 5 neu gemeldeten Infektionen pro Ortsteil und Kalenderwoche zensiert.

Die zweite Datenquelle basiert auf den räumlichen Sozialdaten auf Ortsteilebene, die vom Statistischen Landesamt Bremen bereitgestellt wurden (Datenquelle: Erhebungen des Statistischen Landesamtes, Einwohnermelderegister, Bundesagentur für Arbeit, Senatorin für Kinder und Bildung Bremen). Sie enthielten als Schlüsselfaktoren neben demografischen Informationen auch Daten zu Haushaltsstruktur, Sozial- und Migrationsstatus, Haushaltseinkommen und Sprachförderbedarf im Vorschulalter. Sozialdaten lagen für die Jahre 2020 und 2021 vor. Die letzte Erhebung des Haushaltseinkommens auf Ortsteilebene stammte aus dem Jahr 2013.

Für die räumlichen Analysen wurden die wöchentlichen COVID-19-Inzidenzen als Zeitreihe dargestellt und in 5 Wellen unterteilt. Die daraus berechnete Periodenprävalenz (Anteil an Personen, die innerhalb eines festgelegten Zeitraums an einer Erkrankung leiden) wurde für jede Welle kartografisch dargestellt. Zusätzlich wurde für jede Welle die räumliche Autokorrelation mittels Morans I berechnet, um eine mögliche Clusterbildung zu testen. Morans I ist eine Teststatistik zur Quantifizierung der Ähnlichkeit von Zielgrößen zwischen benachbarten räumlichen Einheiten, die Werte wie ein üblicher Korrelationskoeffizient zwischen −1 (negative Korrelation; diffus) bis 1 (positive Korrelation; geclustert) annimmt. Des Weiteren wurde eine kartografische Übersicht der COVID-19-Periodenprävalenz (pro 1000 Einwohner) pro Welle auf Ortsteilebene erstellt und die Zeitreihe der COVID-19-Inzidenzen je KW und Ortsteil in Boxplots dargestellt, um die räumlichen Unterschiede in den Inzidenzen über die Zeitreihe abzubilden.

Schlüsselfaktoren, von denen angenommen wird, dass sie für die COVID-19-Periodenprävalenz relevant sind, wurden deskriptiv betrachtet. Für eine Untersuchung der Assoziation der Faktoren mit der COVID-19-Periodenprävalenz pro 1000 Einwohner pro Welle wurden lineare Regressionen modelliert. Aufgrund der Vielzahl an Faktoren und möglicher Multikollinearität zwischen diesen wurde eine Variablenselektion durchgeführt, bei der aus dem Modell mit allen Faktoren diejenigen mit der kleinsten Assoziation (*p*-Wert > 0,5) schrittweise ausgeschlossen wurden. Für eine vorwiegend explorative Betrachtung der Faktoren wurde das Signifikanzniveau auf α = 0,1 festgelegt. Es ist darauf hinzuweisen, dass die hier generierten Ergebnisse Assoziationen darstellen, aber noch keine Erklärungen im Sinne einer Kausalität liefern können.

Die räumlichen Analysen und Karten wurden mit R 4.3.1 [25] mit den Paketen sp (2.2.0) und sf (1.0–19) erstellt, während die Regressionsmodelle mit SAS 9.4 (SAS Institute Inc., Cary, NC, USA) berechnet wurden.

## Ergebnisse

**Abb. 1:**
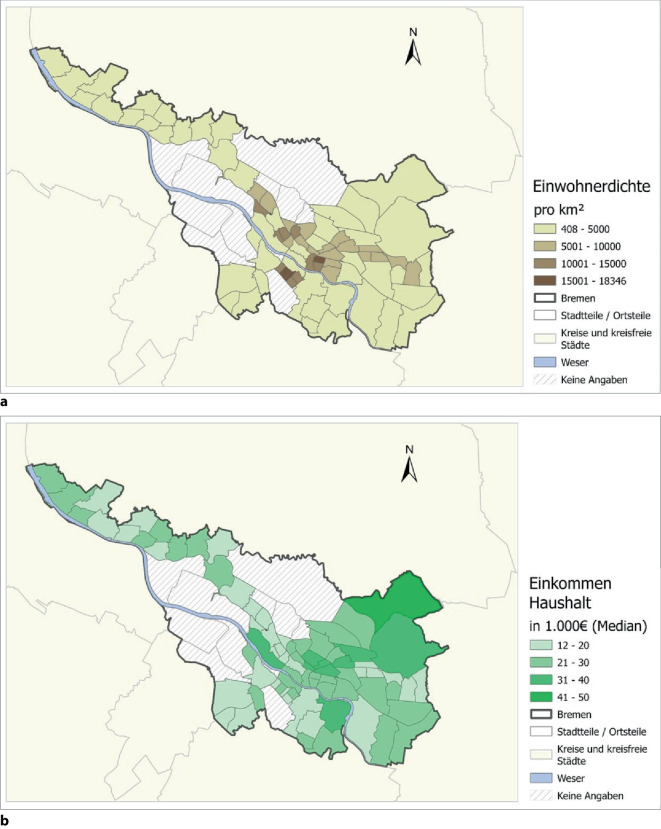
Räumliche Verteilung der Einwohnerdichte (Einwohner pro km^2^) (**a**) und des mittleren Haushaltseinkommens (in 1000 €) (**b**) der Stadt Bremen. Datenquelle: Statistisches Landesamt Bremen, Senatorin für Gesundheit, Frauen und Verbraucherschutz der Freien Hansestadt Bremen

Der finale raumzeitliche Datensatz umfasst 77 der 88 Ortsteile in Bremen mit Zeitintervallen von 117 Wochen beginnend ab dem 02.03.2020 bis zum 23.05.2022 mit verfügbaren und nicht zensierten COVID-19-Inzidenzen je Ortsteil und Kalenderwoche. Die räumliche Verteilung der Bevölkerungsdichte und des mittleren Haushaltseinkommens (Median) ist in Abb. 1 dargestellt. Die Bevölkerungsdichte ist in den meisten innerstädtischen Ortsteilen am höchsten. Zum Vergleich ist das Haushaltseinkommen in den Ortsteilen im Osten, Südwesten und Norden geringer als in der Stadtmitte und in nordöstlichen Ortsteilen (Abb. 1 unten).

**Abb. 2:**
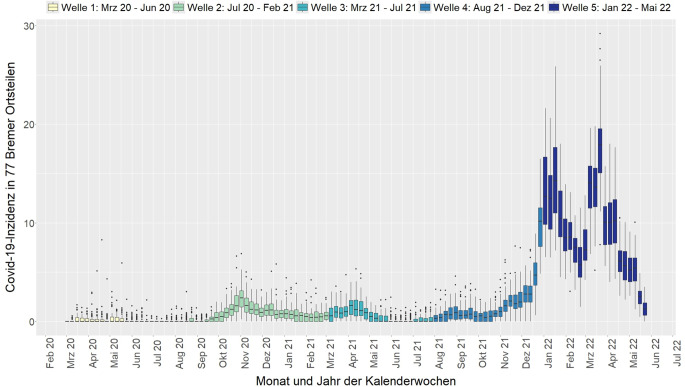
Wöchentliche COVID-19-Inzidenzen in 77 Bremer Ortsteilen im Verlauf der 5 Pandemiewellen (Boxplot). Datenquelle: Statistisches Landesamt Bremen, Senatorin für Gesundheit, Frauen und Verbraucherschutz der Freien Hansestadt Bremen

Die Zeitreihendaten sind in Abb. 2 dargestellt. Die Boxplots veranschaulichen die Verteilung der wöchentlichen COVID-19-Inzidenzen in den Ortsteilen. Bereits in der 1. Welle (März 2020 bis Juni 2020) zeigen sich starke Unterschiede und extrem hohe Inzidenzen in einzelnen Ortsteilen, vor allem im Osten und im Südwesten. Mit dem weiteren zeitlichen Verlauf steigt zwar die durchschnittliche Inzidenz in den entsprechenden Wellen, jedoch weisen einzelne Extremwerte auch auf lokal hohe Inzidenzen hin.

**Abb. 3:**
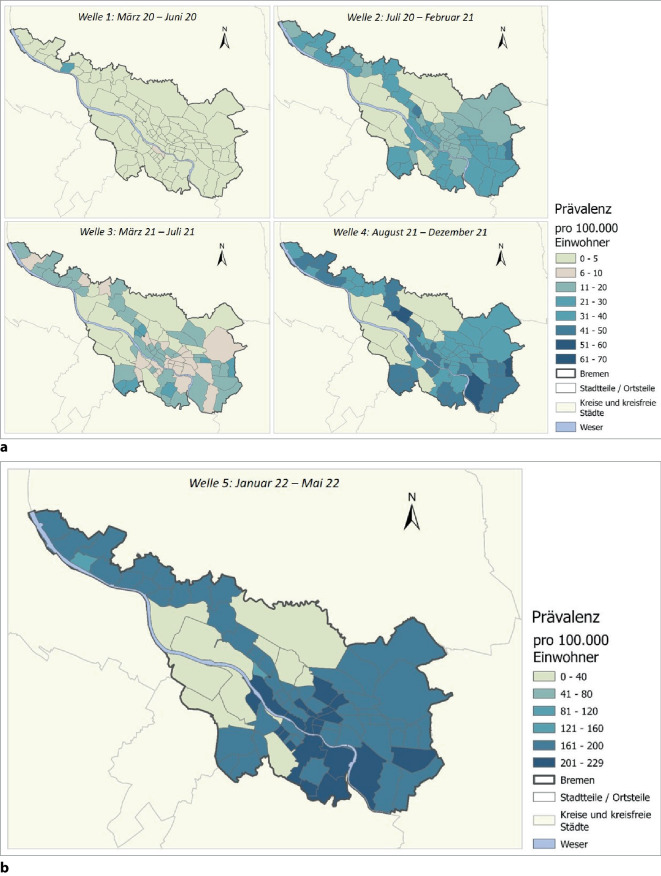
COVID-19-Prävalenz für einzelne Pandemiewellen in der Stadt Bremen. **a** COVID-19-Prävalenz für die Welle 1-4 und **b** Welle 5 pro 100.000 Einwohner in Bremen. Datenquelle: Statistisches Landesamt Bremen, Senatorin für Gesundheit, Frauen und Verbraucherschutz der Freien Hansestadt Bremen

Die kartografische Darstellung in Abb. 3 fasst die COVID-19-Periodenprävalenz für die einzelnen Wellen zusammen. In der 1. Welle gibt es nur vereinzelte Ortsteile mit hoher Periodenprävalenz. In den darauffolgenden Wellen und insbesondere in Welle 4 zeigt sich deutlich eine Clusterbildung in den sozioökonomisch deprivierten Stadtteilen im Osten und Süden, die stärker mit hohen Fallzahlen belastet sind. In Welle 5 (mit veränderter Skala aufgrund sehr hoher Fallzahlen in der Omikron-Welle) sind vorwiegend innerstädtische und sozial bessergestellte Stadtteile betroffen. Tab. 1 zeigt die räumliche Autokorrelation in den einzelnen Wellen. Für die Wellen 2–5 zeigt sich eine signifikante Autokorrelation mit dem höchsten Wert in Welle 4.

**Tab. 1:** Räumliche Autokorrelation der COVID-19-Fallzahlen je Welle in Ortsteilen der Stadt Bremen. Datenquelle: Statistisches Landesamt Bremen, Senatorin für Gesundheit, Frauen und Verbraucherschutz der Freien Hansestadt Bremen

Welle	Zeitraum	Morans I	*p*-Wert*
1	März 2020–Juni 2020	−0,0143	0,51
2	Juli 2020–Februar 2021	0,3008	< 0,001
3	März 2021–Juli 2021	0,3059	< 0,001
4	August 2021–Dezember 2021	0,4634	< 0,001
5	Januar 2022–Mai 2022	0,3197	< 0,001

*Monte-Carlo-Permutationstest (*n* = 999, *p*-Wert)

Eine weitere deskriptive Analyse der Schlüsselfaktoren, die für die COVID-19-Periodenprävalenz relevant erscheinen, zeigt Unterschiede zwischen den 5 Stadtteilen mit den höchsten und den niedrigsten Infektionszahlen pro Welle (Tab. 2). In den Wellen 2–4 wiesen die 5 Ortsteile mit der höchsten Zahl an COVID-19-Infektionen im Vergleich zu den 5 Ortsteilen mit den niedrigsten COVID-19-Infektionen ein geringeres Einkommen, eine geringere Wohnfläche, eine höhere Bevölkerungsdichte und einen höheren Anteil an Kindern mit Sprachförderbedarf im Vorschulalter auf. In Welle 5 waren diese Unterschiede hingegen abgeschwächt oder teilweise invertiert, was auf eine gleichmäßigere Verbreitung von COVID-19 während der Omikron-Welle über die Ortsteile hinweg hindeutet.

**Tab. 2:** Vergleich von soziodemografischen und räumlichen Faktoren in den jeweils 5 Ortsteilen mit den höchsten bzw. niedrigsten COVID-19-Fallzahlen pro Welle in der Stadt Bremen. Datenquelle: Statistisches Landesamt Bremen, Senatorin für Gesundheit, Frauen und Verbraucherschutz der Freien Hansestadt Bremen

	Welle 1: Mrz. 2020–Jun. 2020	Welle 2: Jul. 2020–Feb. 2021		Welle 3: Mrz. 2021–Jul. 2021		Welle 4: Aug. 2021–Dez. 2021		Welle 5: Jan. 2022–Mai 2022	
Je 5 Ortsteile mit …	Höchster Fallzahl	Niedrigster Fallzahl	Höchster Fallzahl	Niedrigster Fallzahl	Höchster Fallzahl	Niedrigster Fallzahl	Höchster Fallzahl	Niedrigster Fallzahl	Höchster Fallzahl
Einwohnerdichte (Einwohner/km^2^), Mittelwert	2926	3460	3607	1479	4969	3518	2964	6395	8562
Median Einkommen (in 1000 €)	20,1	32,3	17,2	34,5	15,3	29,2	18,9	18,2	29,1
Durchschnittl. Wohnfläche (m^2^)	42,0	52,0	36,7	43,0	34,0	50,0	39,0	41,0	47,0
Durchschnittl. Anteil Erholungsfläche (%)	10,8	3,1	12,2	23,9	13,5	24,6	11,3	5,4	3,1
Durchschnittl. Anteil Sprachförderbedarf bei Grundschülern (%)	31,0	9,0	40,0	8,0	42,0	7,0	40,0	22,0	9,0

Die Assoziationen der selektierten Faktoren mit den COVID-19-Fallzahlen pro Welle sind in Tab. 3 zusammengefasst. Für die verschiedenen Wellen zeigen unterschiedliche Faktoren einen Zusammenhang mit der COVID-19-Fallzahl. In der 1. Welle sind ausschlaggebende Deprivationsmerkmale, wie die (sozialversicherungspflichtige) Beschäftigungsquote (β = −0,011, *p* = 0,09), der Anteil der SGBII-Empfänger (β = −0,013, *p* = 0,07) und der Sprachförderbedarf (β = −0,154, *p* = 0,008), negativ assoziiert. In den Wellen 2–4 hingegen zeigt zum Beispiel die SGBII-Quote einen positiven Zusammenhang (Welle 4: β = 0,056, *p* = 0,004), aber die durchschnittliche Anzahl der Kinder pro Haushalt einen negativen (Welle 4: β = −0,061, *p* = 0,039). Der stärkste Zusammenhang findet sich bei der Anzahl der Personen pro Haushalt in Welle 2 (β = 1,099, *p* < 0,001) und 4 (β = 1,51, *p* = 0,012). In Welle 5 hingegen sind nur noch wenige Sozialfaktoren relevant und zum Beispiel ist die Anzahl der Personen pro Haushalt (β = −0,775, *p* = 0,06) negativ assoziiert.

**Tab. 3:** Assoziationen räumlicher Faktoren mit der COVID-19-Periodenprävalenz in 77 Stadtteilen pro Welle im Verlauf der Corona-Pandemie. Datenquelle: Statistisches Landesamt Bremen, Senatorin für Gesundheit, Frauen und Verbraucherschutz der Freien Hansestadt Bremen

	Welle 1: Mrz. 2020–Jun. 2020		Welle 2: Jul. 2020–Feb. 2021		Welle 3: Mrz. 2021–Jul. 2021		Welle 4: Aug. 2021–Dez. 2021		Welle 5: Jan. 2022–Mai 2022	
*Faktor*	β	*p*	β	*p*	β	*p*	β	*p*	β	*p*
Beschäftigungsquote am Wohnort, % (2020)^a^	−*0,011*	*0,09*	0,007	0,20	−0,004	0,49	*0,04*	*<0,001*	–	–
Anteil dt. Staatsbürger mit Migrationshintergrund, % (2020)	−0,008	0,17	*0,008*	*0,025*	0,004	0,3	*0,019*	*0,012*	*0,047*	*0,004*
Anteil SGBII-Empfänger ab 20 Jahren, % (2020)	0,025	0,15	*0,025*	*0,031*	*0,027*	*<0,001*	*0,056*	*0,004*	−0,028	0,23
Anteil SGBII-Empfänger unter 20 Jahren, % (2020)	*−0,013*	*0,07*	−0,007	0,14	–	–	*−0,022*	*0,012*	–	–
Arbeitslosenquote, % (2020)	0,130	0,101	0,065	0,14	*0,121*	*0,035*	–	–	–	–
Durchschnittsalter, in Jahren (2020)	0,016	0,12	–	–	–	–	*−0,045*	*0,004*	*−0,207*	*<0,001*
Anteil ausländischer Staatsbürger, % (2020)	–	–	*0,015*	*0,002*	*–*	*–*	*–*	*–*	*−0,056*	*0,012*
Mittleres Einkommen, Median in 1000 € (2013)	–	–	–	–	−0,006	0,2	–	–	–	–
Anteil Erholungsfläche, % (2014)	–	–	–	–	0,0026	0,1	–	–	–	–
Durchschnittliche Wohnfläche pro Einwohner, in m^2^ (2020)	−0,008	0,24	–	–	–	–	−0,015	0,18	0,038	0,12
Durchschnittliche Anzahl Kinder pro Haushalt (2020)	−0,023	0,25	*−0,039*	*0,007*	*–*	*–*	*−0,061*	*0,039*	–	–
Durchschnittliche Anzahl Personen pro Haushalt	0,366	0,37	*1,099*	*<0,001*	0,085	0,48	*1,51*	*0,012*	*−0,775*	*0,06*
Bevölkerungsdichte (Einwohner/km^2^)	–	–	*0,00001*	*0,02*	7,1*10(-6)	0,27	–	–	–	–
Index (z-Score) Sprachförderbedarf (2018–2020)	*−0,154*	*0,008*	–	–	−0,032	0,36	–	–	–	–

^a^Nur sozialversicherungspflichtige Beschäftigung

Innerhalb von Welle 5 zeigen sich 2 zeitlich getrennte Anstiege in der Inzidenz (Abb. 2), die mit einer Umkehr des sozialen Gradienten einhergehen: Während in der ersten Hälfte von Welle 5 vor allem die benachteiligten Ortsteile höhere Inzidenzen pro Kalenderwoche aufwiesen, waren in der zweiten Hälfte dieser Welle die privilegierten Ortsteile stärker betroffen. Dies unterscheidet sich deutlich von den Wellen 3 und 4, in denen vor allem benachteiligte Ortsteile betroffen waren (Abb. 2 und 3); in Welle 5 traten die höheren Belastungen dagegen eher in innerstädtischen Ortsteilen auf (Abb. 3).

## Diskussion

### Räumliche Unterschiede in den COVID-19-Fallzahlen

Ziel des Beitrags ist es gewesen, einen Einblick in den Zusammenhang von Geografie und Gesundheit (und Krankheit am Beispiel von COVID-19) zu geben und vor diesem Hintergrund das Pandemiegeschehen in Bremen im Längsschnitt auf kleinräumiger Ebene zu analysieren. Es zeigen sich eindeutige räumliche Unterschiede in den COVID-19-Fallzahlen und eine stärkere Belastung von deprivierten Ortsteilen im Verlauf der Pandemie. Dies wird vor allem durch den konsistent positiven Zusammenhang mit der SGBII-Quote in der 2. bis 4. Welle deutlich. Dieser eindeutige Zusammenhang mit der sozialen Lage auf kleinräumiger Ebene wurde in anderen Städten wie Toronto [19], London [26] oder New York [27] festgestellt.

Während die 1. Welle vor allem durch zurückkehrende Reisende geprägt war [28] und in deprivierten Ortsteilen nur wenige Fälle auftraten, stiegen die Fallzahlen in weniger deprivierten Ortsteilen leicht an. Ursachen könnten durch unterschiedliche Möglichkeiten der häuslichen Selbstisolation sowie ungleiche Arbeitsbedingungen zwischen Homeoffice und Dienstleistungsgewerbe bedingt gewesen sein. In den Wellen 2 und 4 zeigte sich ein positiver Zusammenhang der Periodenprävalenz (oder der Anzahl der Infizierten während der Welle) mit der durchschnittlichen Anzahl an Personen pro Haushalt. Das heißt, in Ortsteilen mit höherer Anzahl an Personen pro Haushalt war die Periodenprävalenz pro 1000 Einwohner höher als im Vergleich zu Ortsteilen mit geringerer Anzahl an Personen im Haushalt. Im Vergleich mit Schmiege et al. [21] unterstreicht dies die Bedeutung der Wohnsituation für die Ausbreitung. In Verbindung mit den Erkenntnissen zu den klassischen Deprivationsindikatoren wie SGBII-Quote und Arbeitslosenquote weisen die Ergebnisse damit eindeutig auf die höhere Belastung von Familien in deprivierten Stadtteilen hin. Durch die kartografische Darstellung lassen sich in Bremen insbesondere 3 Cluster im Osten, Südwesten und Norden identifizieren, die auch eindeutig durch höhere Deprivationsindikatoren auffallen.

Wegen der hohen Korrelation vieler Kovariablen und der statistisch gesehen geringen Anzahl an Ortsteilen müssen die Ergebnisse der Regressionsmodelle jedoch mit Vorsicht interpretiert werden. Erstens fehlen wichtige Kontrollvariablen. So wurden die Modelle zwar für das durchschnittliche Alter der Einwohner in den Stadtteilen adjustiert, um die Altersstrukturen zu berücksichtigen, Morbiditätsunterschiede (und Multimorbidität) konnten aber im Modell nicht berücksichtigt werden. Diese könnten gegebenenfalls die Vulnerabilität der Einwohner gegenüber COVID-19 beeinflusst haben und Ergebnisse verfälschen. Zweitens liefern die statistischen Auswertungen keine kausalen Zusammenhänge, sondern nur Assoziationen auf einer ökologischen Ebene. Dieses Problem wird auch als ökologischer Fehlschluss bezeichnet und kann auftreten, wenn Schlussfolgerungen von empirischen Ergebnissen auf der Makroebene auf die Mikroebene (Individuum) übertragen werden [29]. Der direkte Zusammenhang zum Beispiel zwischen der Wohnsituation, individueller Deprivation und den Infektionszahlen ließe sich entsprechend nur über die Erhebung von Individualdaten eindeutig nachweisen [30]. Eine Untersuchung auf individueller Ebene könnte durch räumliche Deprivationsindikatoren unterstützt werden, um den verstärkenden Effekt der Benachteiligung durch die Lebensumwelt zu berücksichtigen (siehe Deprivation Amplification Hypothesis, [31]). Munford et al. zeigten am Beispiel des Vereinigten Königreichs, inwiefern räumliche Benachteiligung je nach Skalierung verstärkende Effekte nicht nur auf die COVID-19-Infektionen, sondern auch auf die Mortalität hatte [32].

Eine weitere Limitation betrifft das Erhebungsjahr für bestimmte Sozialdaten. So stammt die letzte Schätzung des mittleren Einkommens von 2013. Daher wurden zur Darstellung der räumlichen Deprivation mehrere Indikatoren, wie zum Beispiel die Arbeitslosenquote oder SGBII-Quote mit einbezogen, die regelmäßig erfasst werden.

Eine weitere Verzerrung (Bias) in der Analyse der Zeitreihendaten ergibt sich aus veränderten Rahmenbedingungen infolge von Gegenmaßnahmen und medizinischen Entwicklungen. Vor allem die Ausweitung von Schnelltests in späteren Wellen trug zu einer Reduktion der Dunkelziffer bei [33]. Es wird angenommen, dass die Schnelltests im Rahmen der Maßnahmen häufiger in Regionen mit geringer Deprivation genutzt wurden und die Dunkelziffer dort entsprechend geringer ausfiel.

Durch die Lage der Stadt Bremen beziehungsweise die Aneinanderreihung von Ortsteilen entlang der Weser und dem Zensieren der COVID-19-Inzidenzen in gering besiedelten Stadtteilen war es nicht möglich, komplexere räumliche Analysen, wie zum Beispiel geografisch gewichtete Regressionen (Geographically Weighted Regression – GWR; [34, 35]), zu modellieren. Diese Form der lokalen Regression liefert lokal variierende Regressionsschätzer für Zusammenhänge in einzelnen Ortsteilen in Abhängigkeit von Daten in benachbarten Gebieten. Die Ortsteile in der Stadt Bremen orientieren sich jedoch am Fluss Weser und sowohl die benachbarten ländlichen Gebiete als auch die innerstädtischen zensierten Ortsteile führen zu einem lückenhaften Datensatz. Lokale Regressionsmodelle (GWR) beruhen damit zu stark auf sogenannten Grenzeffekten (Edge Effects), um für fehlende benachbarte Werte zu kontrollieren, was zu einem erhöhten Bias in den Modellen führt.

Neben lokalen Regressionen ist auch eine detailliertere Untersuchung der Zeitreihendaten in Verbindung mit der räumlichen Verteilung von Interesse. Eine flexible Möglichkeit der Modellierung bieten gemischte Regressionsmodelle, mit denen eine Varianzkorrektur für geografische Ebenen und wiederholte Messungen modelliert werden kann, um wiederholte Inzidenzen pro KW in Stadtteilen als Endpunkt zu betrachten. Damit lassen sich weitere zeitliche oder auch raumzeitliche Faktoren, wie Temperatur und Niederschlag [36], Luftverschmutzung [37], einbeziehen, die in Assoziation mit dem Infektionsgeschehen stehen. Temperatur und Niederschlag sind insofern von Bedeutung, da sie das Überleben des Virus in der Luft oder auf Oberflächen und zudem den Aufenthalt in geschlossenen Räumen beeinflussen [36]. Luftschadstoffe (z. B. Feinstaub) können die Atemwege reizen und empfänglicher für die Virusaufnahme machen. Zudem kann Feinstaub als Träger des Virus dienen oder die Verweildauer in der Luft erhöhen [37]. Neben den genannten Faktoren können auch die Stärke der gesetzlichen Schutzmaßnahmen auf Landesebene, erfasst über den Stringency Index [38], sowie lokale Impfraten zur Darstellung des Impfschutzes in der Bevölkerung in die Untersuchung von räumlichen und zeitlichen Erklärungsfaktoren einbezogen werden.

Das Beispiel der COVID-19-Pandemie in Bremen verdeutlicht die raumzeitliche Dynamik des Pandemiegeschehens auf kleinräumiger Ebene in Abhängigkeit von kompositionellen und kontextuellen Faktoren.

### Erklärungsmodelle für kleinräumige Variationen

Zur Einordnung und Interpretation der Studienergebnisse können konzeptionelle Ansätze und Erklärungsmodelle kleinräumiger Variationen hilfreich sein. Anzumerken ist, dass diese hier nur kurz und stark vereinfacht beschrieben werden können.

Macintyre et al. (2002) definieren die folgenden 5 Typen von kleinräumigen Merkmalen, die einen gesundheitsfördernden oder gesundheitsschädlichen Einfluss haben können [6]:

1. physische Merkmale, die von allen Bewohnern geteilt werden,
2. Verfügbarkeit von gesundheitsförderlichen Umwelten der Wohnumgebung oder am Arbeitsplatz,
3. öffentliche und private Dienstleistungen, die Menschen im täglichen Leben unterstützen,
4. soziokulturelle Merkmale einer Nachbarschaft sowie
5. Reputation der Wohnumgebung.

Die ersten 3 dieser Merkmale lassen sich nach den Autor:innen auch als materielle oder infrastrukturelle Ressourcen und „Chancenstrukturen“ beschreiben, die die Gesundheit entweder direkt oder indirekt fördern oder beeinträchtigen können. Ein Beispiel für einen direkten Effekt wäre, wenn in Quartieren mit besonders verschmutzter Luft die Gesundheit der Bewohner beeinträchtigt wird und damit die Anzahl schwerer Verläufe von COVID-19 im Vergleich zu anderen Quartieren erhöht ist [36]. Ein Beispiel für einen indirekten Effekt wäre die Verfügbarkeit von Gesundheitseinrichtungen, wie etwa Apotheken, in denen Masken für den Infektionsschutz erhältlich sind. Die letzten beiden Kategorien dieser Typologie beziehen sich auf kollektive soziale Funktionen und Praktiken (z. B. Nachbarschaftshilfe, Austausch von Ressourcen wie Desinfektionsmittel oder Lebensmittel; [39]).

Bernard et al. (2007) haben basierend auf dem Ansatz der „Chancenstrukturen“ ein Konzept zu Nachbarschaft entwickelt [40]. Demnach zeichnen sich Nachbarschaften durch spezifische Ressourcenverteilungen aus, die für die Bewohner etwa zur Wissensgenerierung erforderlich sind (z. B. Wissen zu Übertragungswegen und Schutz vor COVID-19) sowie zur Sicherung des Lebensunterhalts oder zum Aufbau sozialer Beziehungen. Diese werden als sozial determinierte Faktoren beschrieben, die die Gesundheit beeinflussen [4]. Daran anknüpfend definieren die Autor:innen Nachbarschaft als ein soziales System, das sich aus 5 Ressourcendomänen zusammensetzt, die jeweils positive oder negative Ausprägungen annehmen können. Dazu gehören die physische Domäne (z. B. Grünflächen), die ökonomische Domäne (z. B. Supermärkte, Apotheken) sowie die institutionelle Domäne mit öffentlich bereitgestellten Dienstleistungen (z. B. Schulen, Arztpraxen). Darüber hinaus werden die Domäne der Gemeindeorganisationen (z. B. Impfkampagnen) sowie die Domäne lokaler Geselligkeit beschrieben, die kollektive Gruppierungen (z. B. politische Aktivitäten) und sozialen Beziehungen (z. B. Reduzierung von Kontakten) umfassen [39].

Aufbauend unter anderem auf den Ansätzen von Macintyre et al. (2002) und Bernard et al. (2007) entwickelten Voigtländer et al. (2012) weniger einen konzeptionellen Rahmen, sondern ein Erklärungsmodell zum Zusammenhang von Lebenslage, kleinräumigem Kontext und Gesundheit auf 3 Ebenen [11]:

- Lebenslage (Makroebene),
- kleinräumiger Kontext (Nachbarschaft und Region; Mesoebene) sowie
- Verinnerlichung der Ressourcen und Belastungen (Mikroebene).

Die Ebenen sind primär hierarchisch zu verstehen: Die Makroebene (z. B. Beruf, Einkommen) beeinflusst die Mesoebene, also den kleinräumigen Kontext (z. B. physische Umwelt, Arbeitslosigkeit), durch die spezifische Zusammensetzung der Bevölkerung, die wiederum die Mikroebene (z. B. individuelle Wahrnehmung und Bewertung, Verhalten) und letztlich den individuellen Gesundheitszustand prägt. Im Modell sind die Ebenen und Prozesse im Sinne von Ursache-Wirkungs-Beziehungen zu verstehen: Ein hohes Einkommen beeinflusst beispielsweise den gewählten Wohnort und damit den Zugang zu Ressourcen (z. B. medizinische Versorgung, Grünflächen) und damit letztlich auch den individuellen Gesundheitszustand. Auch dieser Ansatz kann als Hilfestellung dienen, um beispielsweise die räumliche Inanspruchnahme von COVID-19-Impfungen vor dem Hintergrund sozialer Ungleichheit (z. B. niedrigere Inanspruchnahme in sozial benachteiligten Quartieren) zu beschreiben. Das Modell verdeutlicht, dass der sozioökonomische Status die gesundheitliche Vulnerabilität maßgeblich beeinflusst und damit ein zentraler Faktor bei der Planung von Gesundheitsmaßnahmen (z. B. Impfkampagnen oder Aufklärungsinitiativen) ist. Alle 3 beschriebenen Ansätze können somit als Hilfestellung zur Beschreibung des kleinräumigen Pandemiegeschehens, hier am Beispiel von Bremen, dienen. Anzumerken ist jedoch, dass das Wissen über die (kleinräumigen) Ursachen und die Dynamik des Pandemiegeschehens, vor allem auf Individualebene, nach wie vor lückenhaft ist, sodass die Ansätze weniger als Erklärung, sondern vielmehr als Unterstützung bei der Interpretation vorliegender Ergebnisse zu verstehen sind.

## Fazit

Die gesundheitsgeografische Perspektive auf das Pandemiegeschehen generiert Erkenntnisse, die sowohl für das Verstehen der Pandemiedynamik als auch für die Implementierung von Maßnahmen im Bereich Prävention oder Bevölkerungsschutz wertvoll sind. Letzteres kann beispielsweise die Durchführung gezielter Impf- oder Informationskampagnen in besonders vulnerablen Stadtteilen sein. Zum Beispiel wurde die Impfkampagne in Bremen unter anderem durch mehrsprachige Informationsschreiben und die lokale Verfügbarkeit des Impfangebots durch einen Impfbus in deprivierten Stadtteilen unterstützt. Ordnungsrechtliche Maßnahmen wie das am 24.03.2020 vom Land Bremen erlassene Kontaktverbot (u. a. Verbot von Veranstaltungen, Zusammenkünften und der Öffnung bestimmter Betriebe) sollten den Anstieg der Infektionsraten eindämmen [41]. Anzumerken ist jedoch, dass die Wirksamkeit der Maßnahmen, auch im Hinblick auf deprivierte Quartiere, bislang noch nicht vollends geklärt werden konnte. Dies ist auch damit zu begründen, dass die Umsetzung der Maßnahmen (z. B. Kontaktverbote) nur schwer zu kontrollieren ist.

Von besonderer Bedeutung für das Verständnis solcher Pandemien ist die Kooperation von Wissenschaft mit dem (kommunalen) Öffentlichen Gesundheitsdienst (ÖGD), die bereits in vielen Fällen regionale Analysen durchführen [42]. Durch die mögliche kleinräumige Verfügbarkeit von Daten sowie die örtliche Expertise könnten für Gesundheitsfragen (ob im Pandemiefall oder bei anderen großen Herausforderungen wie den Folgen des Klimawandels) auf kleinräumiger Ebene Interventionen oder Präventionsmaßnahmen geplant und implementiert werden. Darüber hinaus ist diese Perspektive zur Identifizierung vulnerabler Gruppen in der Bevölkerung notwendig.

Zusammenfassend lässt sich festhalten, dass die räumliche Perspektive auf Gesundheit (und Krankheit), insbesondere im Kontext von Public Health, verstärkt Anwendung finden sollte. Dafür ist es weiterhin erforderlich, den Zugang und die Anwendbarkeit von kleinräumigen Gesundheitsdaten zu vereinfachen.

## Data Availability

Die räumlichen Statistiken und Daten sind beim „Statistischen Landesamt Bremen“ öffentlich verfügbar. Die wöchentlichen kleinräumigen Inzidenzen wurden im Rahmen des Projektes von der Senatorin für Gesundheit, Frauen und Verbraucherschutz der Freien Hansestadt Bremen zur Verfügung gestellt und sind nicht öffentlich verfügbar.
